# Pelvic mass causing hematospermia: splenosis

**DOI:** 10.1186/s12894-022-01138-w

**Published:** 2022-11-16

**Authors:** Yu Jiang, Lei Chen, Min Wang, Xiaocan Li, Dongdong Xie, Dexin Yu, Yi Wang

**Affiliations:** 1 Department of Urology, Second Affiliated Hospital of Anhui Medical University, Hefei, 230601 China; 2 Department of Radiology, Second Affiliated Hospital of Anhui Medical University, Hefei, 230601 China; 3 Department of Pathology, Second Affiliated Hospital of Anhui Medical University, Hefei, 230601 China

**Keywords:** Hematospermia, Splenosis, Splenectomy, Laparoscopy, Obstruction

## Abstract

**Background:**

Most patients with splenosis have no clinical symptoms and do not need intervention. Hematospermia and testicular pain occurred in this patient, which was considered to be related to the huge pelvic implantation of the spleen, which was relatively rare in clinical practice, so we hereby report this case.

**Case presentation:**

A 28-year-old male patient with a history of splenectomy was admitted to the Urology Department of the Second Affiliated Hospital of Anhui Medical University with the chief complaint of "Hematospermia for 1 month and testicular pain for 2 days". Preoperative imaging examination indicated pelvic mass. Combined with the patient's history of splenectomy for splenic rupture in childhood, the possibility of pelvic spleen implantation was considered. Laparoscopic pelvic exploration was performed. During the operation, multiple grayish-brown nodular tissues were observed in the space between the posterior bladder and rectum, and a lobulated grayish-brown mass with a diameter of about 9 cm was observed in the posterior upper part of the prostate gland and seminal vesicle at the pelvic floor. Two nodular tissues were removed intraoperatively and sent for quick frozen pathology, which was reported as spleen tissue. Further resection of the huge mass was performed, and the postoperative pathological results were consistent with the diagnosis of splenosis.

**Conclusion:**

We report a rare case of splenosis presenting with hemospermia and testicular pain.

## Background

Splenosis is a common benign lesion, which is usually found incidentally. After splenic rupture or splenectomy, the splenic fragments or splenic cells spread to the abdominal cavity or adjacent organ tissues. These cells or tissues are implanted, and they receive blood supply from adjacent tissues and gradually differentiate into mature splenic tissues [1–3]. Due to limited blood supply, most implanted spleens are nodular and smaller than 3 cm in diameter, often do not need surgical intervention. However, in a small number of patients, the implanted spleen showed tumor-like growth with a diameter of up to 10 cm [4, 5]. Surgical treatment is usually required when a large implant or a specific implant site causes nonspecific abdominal pain, hydronephrosis, gastrointestinal bleeding, or intestinal obstruction [3, 6]. Herein, we report a rare case of a young male patient who presented with hematospermia for a month and recent testicular pain. Both color ultrasonography and pelvic MRI indicated that a large mass in the pelvic cavity was adjacent to the seminal vesicle. The seminal vesicle endoscopy revealed chronic inflammatory changes in the right seminal vesicle without obvious tumor and stones, which ruled out the possibility of the origin of the seminal vesicle tumor. Combined with the patient's history of splenectomy for splenic rupture in childhood, the possibility of pelvic spleen implantation was considered. Laparoscopy revealed multiple nodules in the pelvic cavity and a splenic-like mass of about 9 cm in the pelvic floor. The histological and pathological results of the excised tissue showed splenic tissue, which was consistent with the diagnosis of splenosis.

## Case presentation

**Fig. 1:**
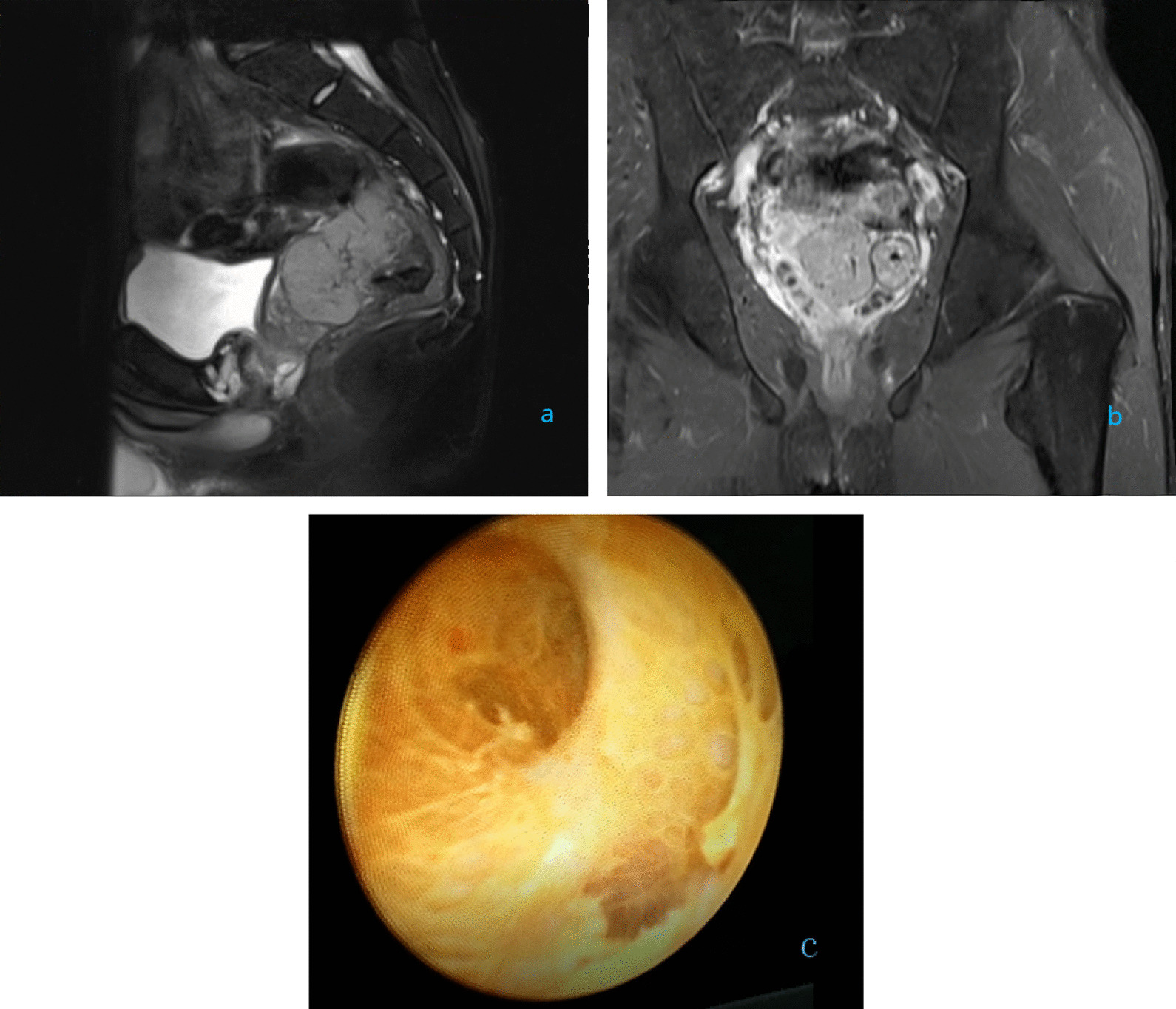
**a**, **b** Pelvic MRI plain scan + DWI + enhancement indicated abnormal signal above the posterior to the seminal vesicle, which was closely related to the seminal vesicle; **c** transurethral seminal vesiculoscopy revealed chronic inflammatory changes in the right seminal vesicle

The 28-year-old male patient was admitted to the Department of Urology of the Second Affiliated Hospital of Anhui Medical University on December 24, 2020 due to "hemospermia for 1 month and testicular pain for 2 days". The patient began to ejaculate with occasional bloody semen 1 month before admission. Right testicular pain started two days before admission. The patient underwent open splenectomy due to rupture of the spleen 20 years ago, and no other past medical history was found. Color ultrasonography of prostate, seminal vesicle, spermatic cord, testis and epididymis indicated enlargement of right seminal vesicle and enhanced echo. The left seminal vesicle gland was normal, and the bilateral ejaculatory duct was not significantly dilated. There was a 74 * 53 mm cystic mass with poor echo posterior to the right side of the prostate. The right epididymis was enlarged and the echo was decreased. There was a dense speckled echo in the right epididymis with abundant blood flow. Pelvic MRI plain scan + DWI + enhancement indicated abnormal signal above the posterior to the seminal vesicle, which was closely related to the seminal vesicle, considering the origin of the seminal vesicle and the possibility of neoplastic lesions. The right seminal vesicle gland was slightly enlarged and the T2W1 signal decreased (Fig. 1a, b).

**Fig. 2:**
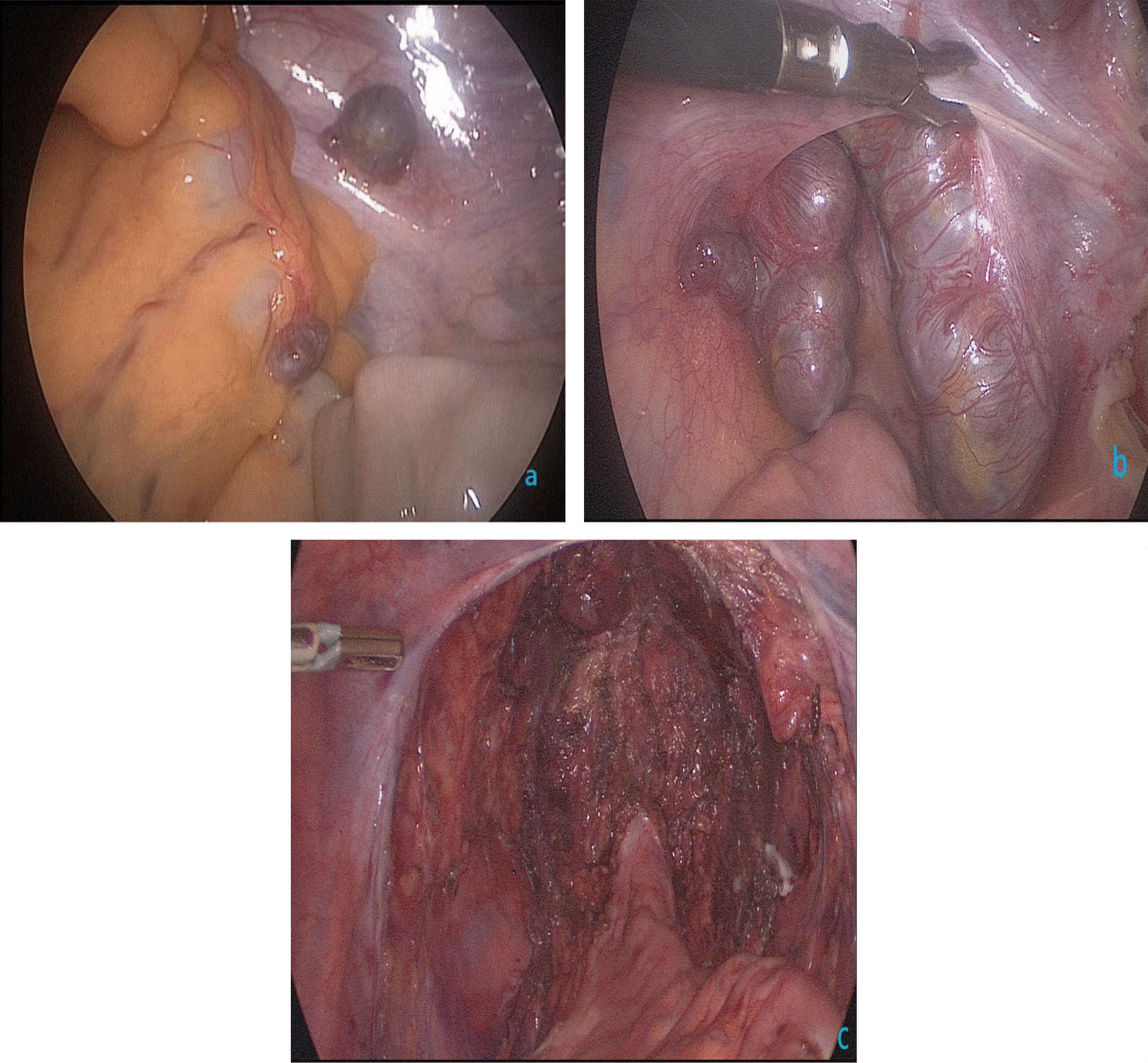
**a**–**c** Laparoscopic pelvic exploration: **a** multiple grayish-brown nodular tissues which was about 1.5 cm in diameter were observed in the pelvic cavity; **b** a lobulated grayish-brown mass with a diameter of about 9cm was observed in the posterior upper part of the prostate gland and seminal vesicle at the pelvic floor; 2c pelvic wound after mass excision

**Fig. 3:**
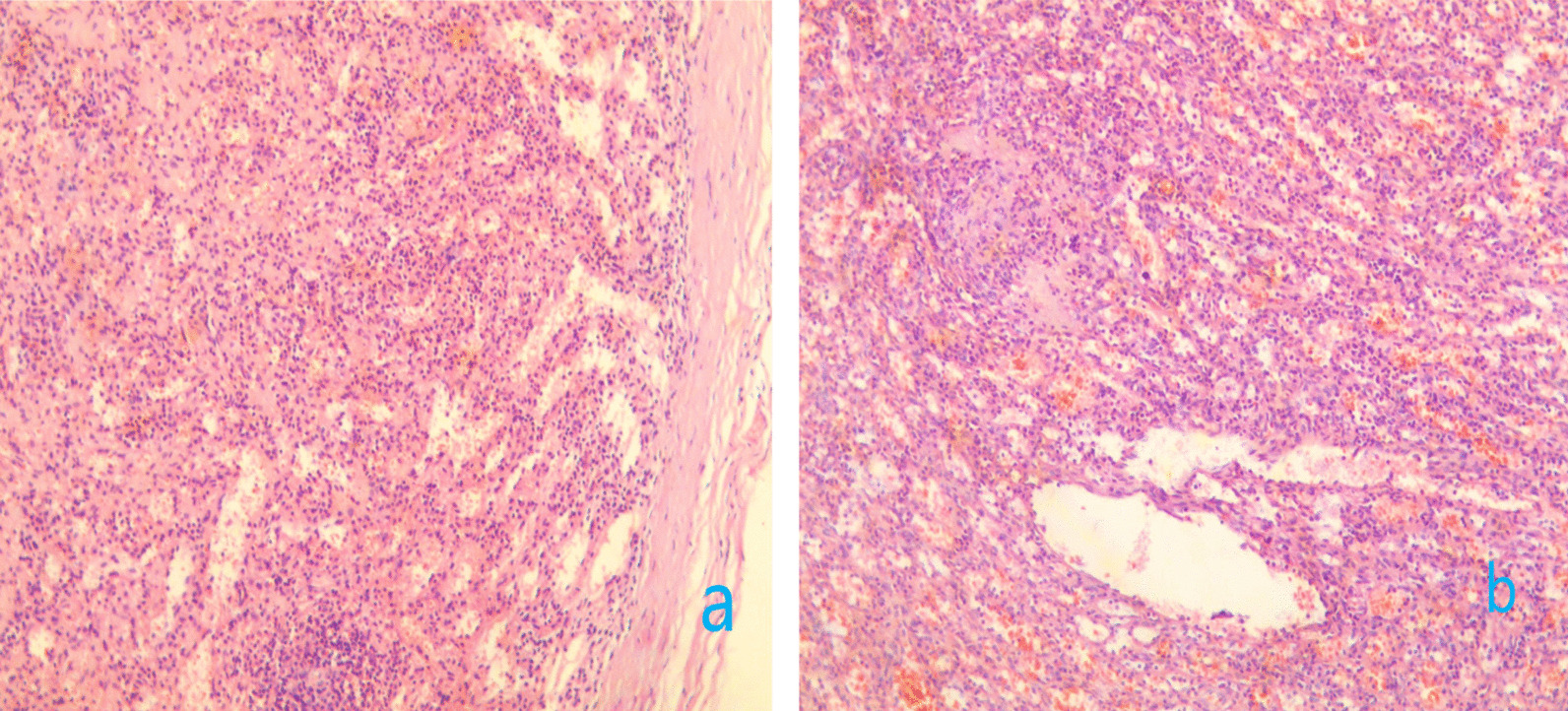
**a**, **b** Pathology: **a** rapid freezing of pathological results during the operation; **b** the postoperative pathological result (HE 40 × )

Although the blood supply of most implanted spleen is limited, the pelvic mass of this patient was large, which did not rule out the possibility of abundant blood supply. Preoperative blood preparation was actively conducted. The patient had a history of open surgery, but considering that it has been 20 years since the last operation, the intraperitoneal adhesions have been absorbed and the operation was mainly performed on the upper abdomen at that time, and the adhesions in the lower abdominal organs may not be serious. In addition, laparoscopic technique has the advantages of clear surgical field, less bleeding, less trauma to patients, less postoperative complications and quick postoperative recovery, so we choose to perform this operation under laparoscopy [7]. It was under general anesthesia that the operation was performed. Before laparoscopic surgery, transurethral seminal vesiculoscopy was performed to further clarify the relevant situation. Verumontanum was identified using a small-diameter ureteroscope (6/7.5-Fr, Wolf, Germany), and the bilateral ejaculatory ducts were then examined. In addition to chronic inflammatory changes in the right seminal vesicle, microscopic examination demonstrated no tumor and calculi, and bilateral ejaculatory ducts were unobstructed (Fig. 1c). Laparoscopic pelvic exploration was performed. During the operation, multiple grayish-brown nodular tissues which was about 1.5 cm in diameter were observed in the pelvic cavity (Fig. 2a), and a lobulated grayish-brown mass with a diameter of about 9 cm was observed in the posterior upper part of the prostate gland and seminal vesicle at the pelvic floor (Fig. 2b). Two nodular tissues were removed intraoperatively and sent for quick frozen pathology, which was reported as spleen tissue (Fig. 3a). Further resection of the huge mass was performed (Fig. 2c), and the postoperative pathological results were consistent with the diagnosis of splenosis (Fig. 3b).

## Discussion and conclusions

Splenosis occurs after rupture of the spleen or splenectomy [8], and its essence is heterotopic autologous splenic tissue implantation [2–4]. Splenosis was first reported by Albrecht [9] in 1896, and Buchbinder and Lipkoff [10] named this disease in 1939. Splenosis occurs in 26%-67% of patients with a history of splenic rupture and splenectomy [4, 5, 11, 12]. In general, splenosis grows slowly and rarely causes invasion. Patients usually have no discomfort symptoms, and it is often accidentally found in physical examination, imaging examination, abdominal surgery and other cases [2, 4]. Spleen tissue or cells can be transplanted not only in various parts of the abdominal cavity [4, 6, 8, 13–15], but also in the retroperitoneal cavity, the chest and even the brain [1, 3, 6, 16–18]. There have been reported cases of spleen transplantation in the kidney and adrenal gland [17, 18]. Most asymptomatic spleen implants do not need the treatment. When obvious enlargement of the implanted spleen or special implant site causes hydronephrosis, intestinal obstruction, gastrointestinal bleeding and other corresponding symptoms, active treatment such as surgical resection is required [3, 6]. Splenosis lacks specificity in clinical imaging examinations such as ultrasound, CT and MRI, and is often confused with tumor [1, 3, 6]. TC-99M9DRBC and indium-111-labeled platelet are relatively specific imaging examinations for splenosis [1–3, 19, 20]. However, these diagnostic devices are not widely available and the cost of examination is very high, which will bring difficulties for accurate preoperative clinical diagnosis of splenosis. Therefore, for patients with a history of splenic rupture or splenectomy, especially in the case of abdominal and retroperitoneal masses, attention should be paid to the possibility of splenic implantation, and relevant preoperative preparations should be actively made.

Hemospermia is usually caused by inflammation, the most common is seminal vesiculitis, but can also be caused by other reasons such as obstruction or cysts, stones, tumors, etc. [21]. The reported patient presented with hemospermia and testicular pain. Multiple preoperative imaging examinations revealed a large pelvic mass closely associated with the seminal vesicle, which was located posterior to the upper part of the seminal vesicle. Endoscopy of seminal vesicle showed no obvious tumors and stones, and the ejaculatory ducts were unobstructed on both sides, but the right seminal vesicle showed chronic inflammatory changes. Therefore, it was considered that the clinical symptoms of the patient might be related to long-term pelvic mass compression of the seminal vesicle and secondary chronic infection. Combined with the patient's history of splenectomy for splenic rupture in childhood, the possibility of splenosis was not ruled out. The nature of the tumor could no longer be determined by routine preoperative imaging, and further pathological examination or expensive radionuclide scan was needed to determine the nature of the tumor. Considering that the large mass had caused obvious symptoms in the patient and there were clear indications for surgery, it was decided to conduct surgical exploration of the mass and submit the intraoperative frozen section for examination. The appropriate treatment was made according to the pathological results.

Based on this rare case, we made a series of advice, which are summarized as follows: First, the establishment of a good follow-up mechanism for patients after splenectomy or rupture of the spleen is not only helpful to observe the survival of the transplanted spleen, but also to monitor the growth and development of the ecotopic implanted spleen that may cause clinical symptoms [22]. Second, urology surgeons should pay attention to individualized plans and carefully consider various factors when treating patients, and do not ignore any small links. This case once again proves that a small detail may play a decisive role in accurate preoperative diagnosis. Attention should be paid to the information provided by the medical history. In this case, we assumed that this history might be forgotten by the patient due to the patient's young age at the time of splenectomy. We should be able to inquire about the history in detail and help the patient to recall the relevant history based on the scar of abdominal surgery at the time of physical examination or ask the relevant informed person about the relevant history(A case of a woman admitted to the hospital with "ovarian cysts and multiple peritoneal nodules" has been reported. The patient underwent splenectomy due to a traffic accident in childhood, and the surgical history was forgotten when the patient provided the medical history in hospital. The peritoneal nodules were suspected to be malignant nodules after examination, and the surgical resection of the nodules was confirmed by pathology as implantation of spleen [5]). When we have an important medical history, we should not simply write it down but think about the meaning behind it.

For patients with a history of splenic rupture or splenectomy, urologists should pay attention to the individualized plan and comprehensively consider all factors in the diagnosis and treatment of patients, and do not ignore any small link. Especially for patients with abdominal or retroperitoneal masses, attention should be paid to the possibility of splenosis, and we should carefully identify the tumor and avoid excessive treatment.

## Data Availability

All the data supporting our findings are contained within the manuscript.

## Abbreviations

MRI: Magnetic resonance imaging
DWI: Diffusion-weighted imaging
CT: Computed tomography
TC-99M9DRBC: Tc-99m-tagged heat-damaged red blood cells scintigraphy
